# CT-Based Predictor for the Success of 12/14-Fr Ureteral Access Sheath Placement

**DOI:** 10.1155/2022/3343244

**Published:** 2022-11-02

**Authors:** Jieping Hu, Yue Yu, Wei Liu, Jialei Zhong, Xiaochen Zhou, Haibo Xi

**Affiliations:** Department of Urology, The First Affiliated Hospital of Nanchang University, Nanchang, China

## Abstract

**Purpose:**

Ureteral access sheaths (UAS) are widely used in retrograde intrarenal surgery (RIRS), and this study aimed to develop a model for predicting the success of UAS placement based on computed tomography.

**Methods:**

We analyzed the clinical data of 847 patients who received ureteroscopy. Data on patient and stone characteristics and several computed tomography (CT)-based measurements were collected. A nomogram predicting the success of UAS placement was developed and validated using *R* software.

**Results:**

Two hundred and forty-seven patients were identified. Twenty-five patients (10.1%) failed to pass through the UAS. A model with three factors including the short diameter of ureteral calculi, the short diameter of hydronephrosis, and the diameter of the narrowest part of the renal parenchyma was to be strongly practical and had a high area under the curve on internal validation (80.3%). Using a threshold cutoff of 92%, the sensitivity and specificity for predicting UAS placement were 0.35 and 0.92, respectively.

**Conclusion:**

Our study provides a nomogram for predicting the success of UAS placement, and this model could help discriminate patients who are likely to suffer from failed UAS insertion; preoperative ureteral stenting is recommended according to the prediction.

## 1. Introduction

Retrograde intrarenal surgery (RIRS) is one of the first-line options for renal or proximal ureteral stones up to 20 mm [1]. Ureteral access sheaths (UAS) increase visibility, reduce operation time, and allow multiple re-entries to the ureter while reducing intrarenal pressure and providing drainage for irrigation fluids [2]. However, failure rates of ureteroscopy due to a difficult impassable ureter range from 8% to 10%, and approximately, 22% of patients fail to insert a standard UAS [3]. Stent placement before RIRS can theoretically expand the ureter to increase the success rate of UAS placement [4] but entails a two-stage procedure for all patients [3]. So far, European Association of Urology (EAU) guidelines suggest that the routine placement of ureteral stents before RIRS for renal stones is not required [4]; preoperatively identifying patients who need prestenting results in higher success for UAS placement and minimizes intraoperative ureteric injury.

Age, previous same-side procedures, and preoperative stent were indicated to be independent predictors for an effective 14F UAS insertion [3], while others showed that prestenting status was the only independent factor for successful access sheath insertion [5]. Recently, patients with normal BMIs and a tent-shaped ureteral orifice over the introductory guidewires were found to be more likely to achieve primary UAS insertion [6], and male gender and ipsilateral hydronephrosis may be associated with increased UAS insertion failure [7]. Despite this progress, the success of UAS placement is difficult to predict preoperatively. Noncontrast-enhanced computed tomography (CT) can determine the stone size, proximal ureteral diameter, degree of hydronephrosis, and surrounding anatomy, experienced urologists can often judge the success rate of UAS placement from CT, and CT-based tools/measurements were applied to identify key predictors of successful ureteral stone passage [8–10]. Thus, we were enlightened to develop a model for predicting the success of UAS placement based on CT. We hope to include as many parameters as possible for analysis. Based on literature and clinical experience, seven CT parameters were included for analysis, including indicators reflecting stone, ureter, and kidney characteristics.

## 2. Patients and Methods

### 2.1. Study Design and Participants

This retrospective study was approved by the institutional review board of the First Affiliated Hospital of Nanchang University, and it was exempted from obtaining informed consent. We conducted the trial following the principles of the ethical principles of the Declaration of Helsinki. We included adults aged≥18 years old with stones confirmed by noncontrast computed tomography (CT) with a slice thickness of 1 mm. We retrospectively analyzed the medical records and CT of 247 consecutive patients who underwent RIRS between May 2020 and February 2022 at our institution. Patients were screened by the following inclusion and exclusion criteria [10]. The inclusion criteria were as follows: 1 upper ureteral stones confirmed by CT; 2 the patient agreed to receive RIRS (either as a primary intervention or after failed conservative management); 3 12/14-Fr UAS was used; 4 age≥18 yrs. The exclusion criteria were as follows: 1 ureteral multiple-calculus; 2 preoperative ureteral stenting; 3 abnormal urinary tract anatomy (such as horseshoe kidney or ileal conduit); 4 patients received RIRS under local anesthesia; 5 11/13-Fr, 14/16-Fr, or another size of UAS was used; 6 the patient had previously undergone ureterolithotomy; 7 balloon catheter dilation was performed; 8 failure to receive RIRS due to pyonephrosis; 9 the patient was taken CT at another hospital and parameters cannot be measured; 10 patients received RIRS because of renal stones combined with middle or lower ureteral stones.

### 2.2. Statistical Analyses

**Figure 1:**
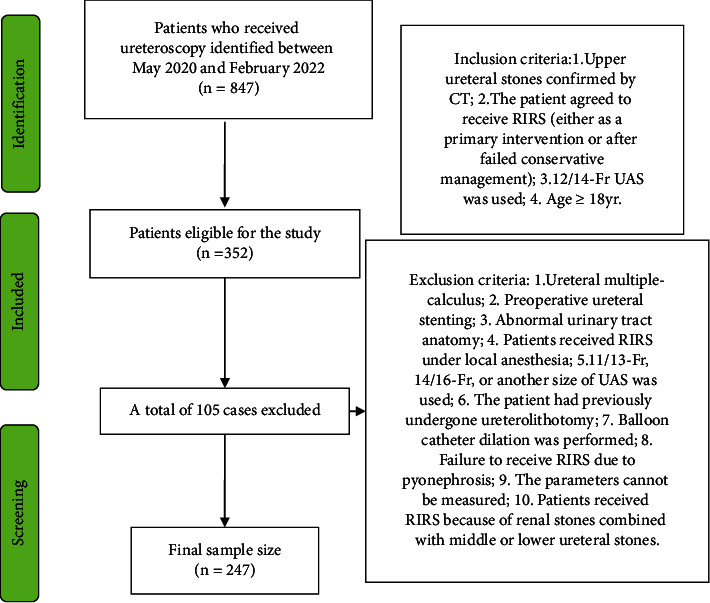
Flow diagram of the patient selection.

The patient and stone characteristics including age, gender, length of history, stone size, side of the ureteral stone, and several CT-based measurements were collected [10]. The CT-based measurements contained seven parameters: ①long diameter of ureteral calculi; ②short diameter of ureteral calculi; ③ureter diameter at about 1 cm above ureteral calculi; ④long diameter of hydronephrosis; ⑤short diameter of hydronephrosis; ⑥diameter of the widest part of the kidney parenchyma; ⑦diameter of the narrowest part of the renal parenchyma (Figure 1).

Continuous variables are expressed as the mean value and standard deviations. Categorical variables are expressed as the frequencies of events (%). Pearson's chi-squared test and *T*-test were used for comparing the two groups. Factors affecting the success of UAS placement were analyzed using univariate logistic regression. Regression coefficients were calculated and used to develop the nomogram. The area under the curve was calculated to evaluate the nomogram's predictive accuracy. The observed UAS insertion rate was described graphically in logistic calibration plots. Last, a decision curve analysis for the model was performed. All statistical tests were performed using IBM SPSS Statistics, version 22.0 (IBM, Armonk, NY, USA), *R* v.3.6.2 (https://www.r-project.org), Stata (version 12.0, StataCorp), and a *p* value <0.05 indicated statistical significance.

## 3. Results

**Figure 2:**
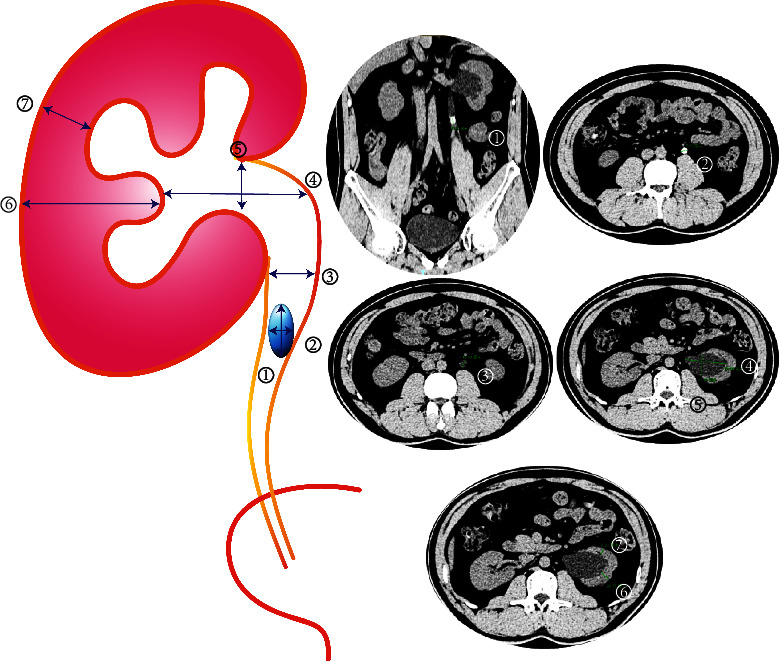
The computed tomography (CT)-based measurements: ① long diameter of ureteral calculi; ② short diameter of ureteral calculi; ③ ureter diameter at about 1 cm above ureteral calculi; ④ long diameter of hydronephrosis; ⑤ short diameter of hydronephrosis; ⑥ diameter of the widest part of the kidney parenchyma; ⑦ diameter of the narrowest part of the renal parenchyma. The CT-based measurements were measured in a typical case: ① = 11 mm, ② = 9 mm, ③ = 10 mm, ④ = 60 mm, ⑤ = 29 mm, ⑥ = 14 mm, and ⑦ = 8 mm; mm: millimeter.

A total number of 847 patients who received ureteroscopy were screened. Six hundred patients were excluded according to inclusion and exclusion criteria; 247 patients were identified for further analysis (Figure 2). Patient data are presented in Table 1. Twenty-five (10.1%) patients failed to perform RIRS due to failure to pass the UAS (group F), and two hundred and twenty-two patients were successful to insert UAS (group S). Age, gender, stone side, and length of history were no differences between the two groups. For CT-based parameters, the short diameter of hydronephrosis was less in group F than in group S (*p*=0.04, Table 1), while other parameters had no significant difference between the two groups.

**Figure 3:**
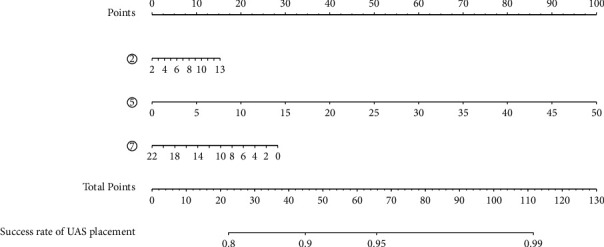
A nomogram predicting the probability of ureteral access sheath (UAS) placement. Instructions: locate the variable value on the corresponding axis. Draw a line straight upward to the point axis to determine how many points are towards the probability of UAS placement that the patient receives for his risk score value. Repeat the process for each addition variable. Sum the points for each of the predictors. Locate the final sum on the total point axis. Draw a line straight down to find the patient's success rate of UAS placement.

**Figure 4:**
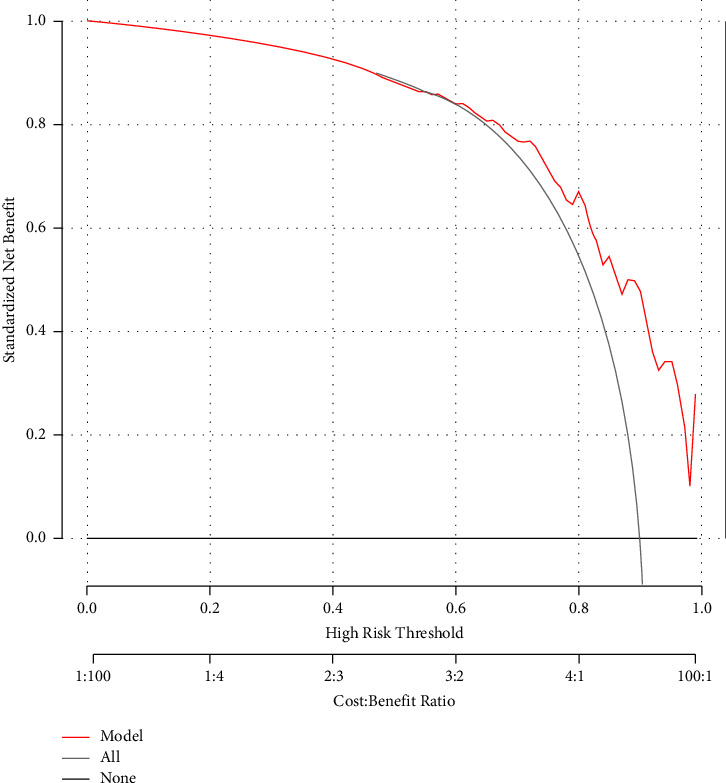
Decision curve analysis of the logistic model.

Univariate logistic regression analysis was performed for factors affecting the success to insert UAS, and none of the parameters were found to be associated with UAS placement (Supplement Table 1). To establish a prediction model of success for sheath, we first included all parameters for model establishment, and the AUC of the model was 0.82. According to the comparative analysis of the two groups and the preliminary model results, in order to achieve a good prediction effect without including too many parameters, we developed several nomograms to predict the success rate of UAS placement and found a model with three factors to be strongly practical and effective (Figure 3). Parameters ② Short diameter of ureteral calculi, ⑤ Short diameter of hydronephrosis, and ⑦ Diameter of the narrowest part of the renal parenchyma were included in the model with a high AUC on internal validation (80.3%).  Table 2 listed systematic analyses of the nomogram-derived cutoffs used to discriminate between patients who received successful or failed UAS placement. Using a threshold cutoff of 92%, the sensitivity and specificity for predicting UAS placement were 0.35 and 0.92, respectively. Thus, 167 (67.6%) patients were recommended to receive prestent, and failed UAS placement would be unpredicted in only two (2.5%) patients (Table 2). The decision curve analysis (DCA) demonstrated that the model had a high clinical net benefit (Figure 4).

## 4. Discussion

Up to 22% of patients who received RIRS would be failed to insert UAS due to orifice shape and angle (not stenosis), narrow ureter, twisted ureter, and duplex ureter [3, 11]. Meta-analysis suggested that prestenting resulted in higher success for UAS placement, minimizing intraoperative ureteric injury [1], and patients always received two-stage RIRS after UAS placement for two weeks when failed to insert UAS for the first surgery. It was of important clinical significance to distinguish between patients who would succeed or fail to insert UAS. Prediction of UAS placement can guide treatment plans, reduce hospitalizations, avoid UAS-related ureteral injuries, and reduce costs, and preoperative ureteral stenting would be performed for the selected patients (who failed to insert UAS) [12]. CT-related parameters had been used as predictors of spontaneous passage of ureteral stones and shock wave lithotripsy success [8, 9, 13], and we analyzed seven relevant indicators and developed a practicable nomogram model for prediction of UAS placement in this study.

To our best knowledge, this is the first study for predicting UAS insertion success. Results showed the short diameter of ureteral calculi, the short diameter of hydronephrosis, and the diameter of the narrowest part of the renal parenchyma were relevant factors. The diameter of the renal parenchyma was negatively correlated with the success of UAS placement, and the diameter of ureteral calculi and hydronephrosis were positive factors. Of these parameters, the diameter of the renal parenchyma and ureteral calculi were not reported to be predictors for an effective 14F UAS insertion. Preoperative measurement of the ureteral diameter was recommended for ureteral access sheath placement to predict the risk of ureteral injury [14], and our model was found to predict the value of the diameter of hydronephrosis instead of ureteral diameter. The applicator should be noted that discrimination was good when the threshold was between 0.86 and 0.91 (Table 2), and if the value is outside the interval, the model's performance will decrease. The size of UAS for RIRS was usually 12/14 Fr in our institution, 10/12 Fr or 14/16 Fr were seldom used, the use of 10/12 Fr UAS had an advantage for the insertion success rate along with the incidence of systemic inflammatory response syndrome increased for stones > 2 cm, prestent was recommended for patients who received RIRS using 14/16 Fr UAS to avoid ureteral injury, and research indicated limiting the insertion force to ≤ 6 N can avert UAS-associated ureteral injury for patients without preoperative indwelling ureteral stent, but so far, tools to monitor the force of sheath placement are not common [16]. What is more, the cut-off value for a recommendation of prestent should balance sensitivity and specificity. We recommended the threshold to be 0.92, 144 of 222 patients with successful UAS placements would receive excessive prestent, and 23 of 25 failed UAS placements were recommended prestent in the model. The higher threshold you postulated, the more patients received overtreatment.

Our model has several advantages. First, the parameters in this model were easy to measure, and clinicians can take measurements without the help of an imaging specialist. Second, the errors of measurement were relatively small, the value would be of high consistency among distinct surveyors, and some parameters may be less reproducible, such as ureteral wall thickness or ureteral jet flows [17, 18]. Third, the 12/14F UAS was considered the universal UAS that accepts all flexible ureteroscopes that are available in the endourology field [19], and this model would be widely used for prediction. Fourth, the model was simple, but with a high AUC value, only three factors were included, the total point was easy to calculate, and the success of UAS placement was also visualized.

Despite several strengths, our study is not devoid of limitations. First, the relatively small sample size and the number of events are to be viewed as a major limitation, only 25 patients failed to insert UAS in the model, which may undermine the accuracy of prediction, and the sample size was big enough to conclude due to restricting inclusion and exclusion criteria. Second, the excellent performance characteristics of our nomogram might be related to the use of internal validation, and formal external validation would be better to convince the model before implementation in clinical practice [20]. Third, our model was developed using data from one institution in China, which represented the Asian population and limited its generalizability to other races. Fourth, our model only included the CT-based parameters, a relationship between relatively low platelet levels and UAS insertion failure was found recently [7], and other clinical parameters may be included for further analysis. Fifth, worldwide use of UAS for renal stones has risen over the last decade [21], the model excluded the patients with renal stones who had undergone RIRS, and only patients with proximal ureter calculus were suitable for using this model. Last but not least, it was a retrospective study, and thus, patient selection biases existed as typical of all retrospective series.

## 5. Conclusions

Our study provides a nomogram predicting the success of UAS placement. Adoption of this model could help discriminate patients who are likely to suffer from failed UAS insertion, preoperative ureteral stenting is recommended according to the prediction, and using a 0.92 threshold would identify 92% of patients to have failed UAS placement.

## Figures and Tables

**Table 1:** Characteristics of patients.

Parameters	Failed to perform RIRS without prestent (*n* = 25)	Success to perform RIRS without prestent (*n* = 222)	*p* value
Age (years), mean (SD)	45.9 ± 12.9	48.0 ± 12.9	0.44
Gender (males), *n* (%)	19 (76.0%)	137 (61.7%)	0.16
Stone side			
Right	12	96	0.65
Left	13	126	
Length of history			
0–14 days	16	123	0.17
15–31 days	6	35	
>31 days	3	64	
CT-based parameters			
Long diameter of ureteral calculi (mm), mean (SD)	9.12 ± 4.38	10.13 ± 4.52	0.29
Short diameter of ureteral calculi (mm), mean (SD)	6.60 ± 2.27	7.13 ± 2.07	0.24
Ureter diameter at about 1 cm above ureteral calculi (mm), mean (SD)	9.12 ± 4.05	10.39 ± 3.93	0.13
Long diameter of hydronephrosis (mm), mean (SD)	36.48 ± 11.59	38.89 ± 11.06	0.31
Short diameter of hydronephrosis (mm), mean (SD)	11.64 ± 6.00	14.48 ± 6.54	0.04
Diameter of the widest part of the kidney parenchyma (mm), mean (SD)	23.40 ± 5.34	21.95 ± 4.95	0.17
Diameter of the narrowest part of the renal parenchyma (mm), mean (SD)	13.20 ± 3.29	12.16 ± 3.44	0.15

RIRS: retrograde intrarenal surgery; SD: standard deviation; mm: millimeter.

**Table 2:** Systematic analyses of the nomogram-derived cutoffs used to discriminate between patients who received successful or failed UAS placement.

Threshold	Patients below the cut-off value (prestent recommended)		Patients above the cut-off value (prestent not recommended)	
	Success	Failed	Success	Failed
0.80	1	0	221	25
0.82	4	0	218	25
0.84	16	6	206	19
0.86	55	11	167	14
0.88	94	15	128	10
0.90	120	19	102	6
0.91	125	21	97	4
0.92	144	23	78	2
0.94	180	23	42	2
0.96	212	24	10	1
0.98	220	24	2	1

UAS: ureteral access sheath.

## Data Availability

The data used to support the findings of this study are available from the corresponding author upon request.

## Supplementary Materials

Supplementary Materials Supplementary Table 1: univariate logistic regression analyzes for factors affecting the success of UAS placement.
