# Supplementary material for: CT-Based Predictor for the Success of 12/14-Fr Ureteral Access Sheath Placement
Source: Int J Clin Pract. 2022 Nov 2;2022:3343244. doi: 10.1155/2022/3343244 (PMC9646298; doi:10.1155/2022/3343244)
Supplement: Supplementary Materials — Supplementary Table 1: univariate logistic regression analyzes for factors affecting the success of UAS placement. [file 3343244.f1.docx]

| Supplement Table 1. Univariate logistic regression analyze for factors affecting the success of UAS placement | | | | |
| --- | --- | --- | --- | --- |
|  | B | SD | P value | Exp(B) |
| Age | 0.008 | 0.017 | 0.659 | 1.008 |
| Sex | -0.721 | 0.519 | 0.164 | 0.486 |
| Sides | 0.089 | 0.452 | 0.845 | 1.093 |
| Long diameter of ureteral calculi | 0.018 | 0.068 | 0.794 | 1.018 |
| Short diameter of ureteral calculi | 0.025 | 0.14 | 0.857 | 1.026 |
| Ureter diameter at about 1cm above ureteral calculi | 0.017 | 0.078 | 0.832 | 1.017 |
| Long diameter of hydronephrosis | -0.032 | 0.031 | 0.298 | 0.968 |
| Short diameter of hydronephrosis | 0.11 | 0.067 | 0.102 | 1.116 |
| Diameter of the widest part of the kidney parenchyma | -0.018 | 0.053 | 0.736 | 0.982 |
| Diameter of the narrowest part of the renal parenchyma | -0.03 | 0.083 | 0.714 | 0.97 |
| Length of history, (0-14d) |  |  | 0.193 |  |
| Length of history, (15-31d) | -0.841 | 0.673 | 0.211 | 0.431 |
| Length of history, (>31d) | -1.39 | 0.767 | 0.07 | 0.249 |
